# Humanity Test—EEG Data Mediated Artificial Intelligence Multi-Person Interactive System

**DOI:** 10.3390/s24247951

**Published:** 2024-12-12

**Authors:** Fang Fang, Tanhao Gao, Jie Wu

**Affiliations:** College of Design and Innovation, Tongji University, Shanghai 200092, China; fangfangtongji@foxmail.com (F.F.); tanhaogao@gmail.com (T.G.)

**Keywords:** electroencephalogram (EEG) data, artificial intelligence, multi-person interaction, emotional experience, medium, installation, brain-computer interface (BCI)

## Abstract

Artificial intelligence (AI) systems are widely applied in various industries and everyday life, particularly in fields such as virtual assistants, healthcare, and education. However, this paper highlights that existing research has often overlooked the philosophical and media aspects. To address this, we developed an interactive system called “Human Nature Test”. In this context, “human nature” refers to emotion and consciousness, while “test” involves a critical analysis of AI technology and an exploration of the differences between humanity and technicality. Additionally, through experimental research and literature analysis, we found that the integration of electroencephalogram (EEG) data with AI systems is becoming a significant trend. The experiment involved 20 participants, with two conditions: C1 (using EEG data) and C2 (without EEG data). The results indicated a significant increase in immersion under the C1 condition, along with a more positive emotional experience. We summarized three design directions: enhancing immersion, creating emotional experiences, and expressing philosophical concepts. Based on these findings, there is potential for further developing EEG data as a medium to enrich interactive experiences, offering new insights into the fusion of technology and human emotion.

## 1. Introduction

The application of artificial intelligence (AI) systems has become increasingly widespread across diverse fields. These systems are employed not only in daily life for intelligent assistants, automated services, and recommendation systems but also play crucial roles in sectors like healthcare, education, and manufacturing [1]. The research focus lies within the field of human-computer interaction, specifically on how to enable machines to effectively comprehend and react to human needs, emotions, and behaviors [2], AI research utilizing physiological data represents an important branch involving physiological signals such as electroencephalograms (EEGs), heart rates, and skin conductance to enhance machine understanding of human emotions and intentions [3]. Current research in this branch varies, with some studies concentrating on AI applications in wearable biosensors [4], others on machine learning algorithms for physiological data analysis [5] for emotion recognition, and additional investigations into AI techniques for managing extensive biometric data sets, processing outliers, and maintaining stability under diverse challenging conditions [6,7].

However, most of these studies focus on the medical and signal-processing fields, with few concentrating on how physiological signals could augment emotional communication and immersive experiences between AI systems and users [8]. This contradicts the current society’s need to understand the interaction between human emotions and machines [9]. Therefore, the specific objective of this study is to explore how multi-user EEG data can enhance immersion, emotional interaction, and the conveyance of concepts in AI systems. We designed a prototype system to adjust and provide feedback on interactive experiences by capturing and analyzing participants’ brainwave activity in real time. This research utilizes experimental approaches and user feedback to assess the system’s effectiveness. Through detailed analysis of participants’ data during interactions, methods to augment experiential and emotional engagements are discussed.

In summary, this study not only establishes a new foundation for improving AI systems’ experiential quality but also aims to build solid foundations for designing AI systems with enhanced emotional intelligence, contributing to the future development of the field of human-computer interaction.

## 2. Theoretical Framework

**Figure 1:**
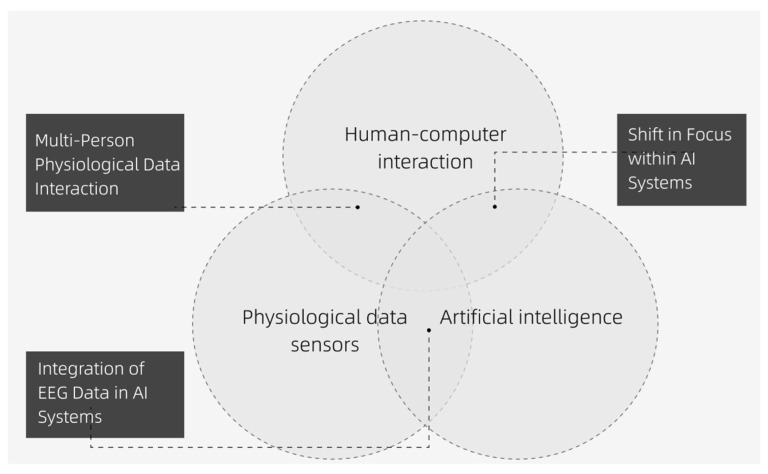
Three intersecting fields of human-computer interaction, physiological data sensors, and artificial intelligence.

For this study, literature was primarily retrieved through Google Scholar, the ACM Digital Library, IEEE Xplore Digital Library, and Springer Link Online Library. Research on integrating multi-user EEG data into AI systems lies at the intersection of human-computer interaction, physiological data sensors, and artificial intelligence. Consequently, most of the relevant publications are found in the fields of human-computer interaction and computer science, with papers mainly published in leading international conferences such as CHI, IEEE VIS, SIGGRAPH, and HCI, as well as journals like Leonardo, Sensors, Entertainment Computing, and Digital Creativity. We first identified a set of keywords and search strings closely related to the research topic, following a search path from “physiological data interaction design” to “multi-user physiological data interaction design”, “multi-user EEG data interaction design”, “AI interaction device design”, and finally “AI systems integrated with EEG data design”. The collected literature was then categorized into three intersecting fields and analyzed from the following three perspectives (see Figure 1): “Shift in Focus within AI Systems”, “Multi-Person Physiological Data Interaction”, and “Integration of EEG Data in AI Systems”.

Employing media theory [10] as a framework, this study examines the shortfall in critically examining AI technology and neglecting immersive and emotional interactions in offline multi-person AI systems. This research emphasizes the potential of multi-user EEG data to improve AI systems’ experiential quality.

### 2.1. Shift in Focus Within AI Systems

In recent years, advancements in machine learning and natural language processing have led to the integration of AI into diverse facets of human-computer interaction and design. This approach opened up new design possibilities, notably enhancing content diversity and unpredictability. Yet, AI’s role is frequently reduced to creating varied visual effects [11], enabling designers to perform more significant technical manipulation. Yet, there is little critical research on AI technology from a media and philosophical perspectives, nor exploration of thoughts on humanity and technicality [12].

**Figure 2:**
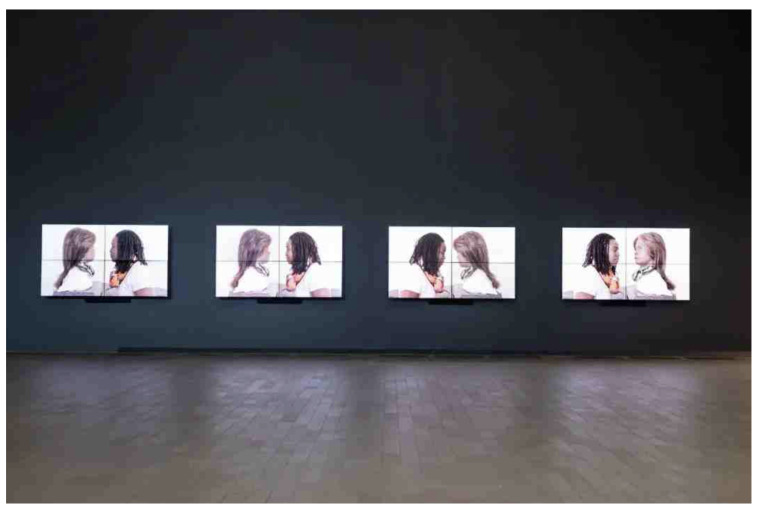
The “Uncanny Valley: Being Human in the Age of AI” exhibition.

McLuhan’s “the medium is the message” principle [10] underscores the significant influence of medium form on societal and individual cognition. Rokeby argued for an exceptional understanding of technology, one that penetrates the philosophy behind it [13]. Considering the philosophical, psychological, political, and even aesthetic aspects behind practical projects is crucial. As shown in Figure 2, the “Uncanny Valley: Being Human in the Age of AI” exhibition reimagines the human-AI relationship from a novel viewpoint [14]. Through abstract interactive works, the exhibition showcases AI’s cognitive biases and creative environments, along with the issues and impacts arising in contemporary life. The “Deep Feeling: AI and Emotions” exhibition explores emotions and new self-awareness through the gradual symbiosis between humans and AI [15]. Antonio Daniele et al. leveraged the “Re: Humanism” movement as an artistic Turing test, prompting reflection on human essence and perceptions of artificiality by distinguishing between what is inherently human and what is fabricated [16].

**Figure 3:**
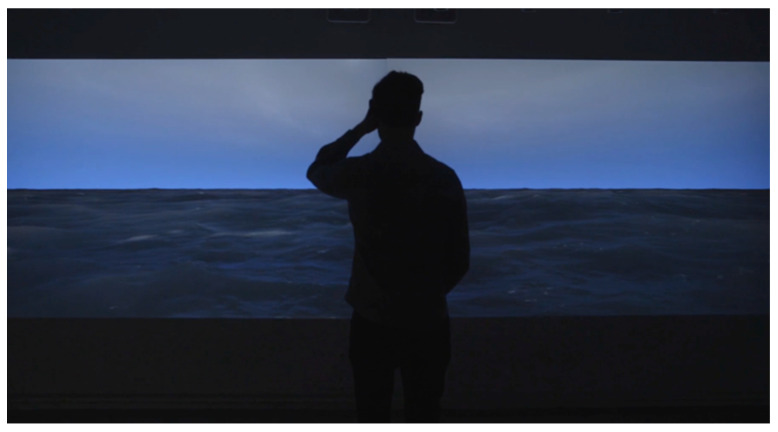
Interface Interaction.

Recent studies have revealed that AI interaction systems’ outputs are predominantly interface-based, displaying information directly on screens for viewers. This primarily visual mode offers an instantaneous experience, involving viewers interacting with the screen in front of them through clicks (see Figure 3). However, this approach has diminished user experience, prompting researchers to explore more immersive output modes for AI systems. For instance, an AI-enabled device for writing Chinese characters set within a virtual bamboo forest merges calligraphy and painting, offering an immersive experience that blends reality with transcendence [17]. Cacophonic Choir, an interactive art installation, employs a neural network trained on sexual assault narratives to craft an immersive auditory and visual experience, highlighting survivors’ stories [18]. AI systems facilitate improvisational practice in theater settings, enabling actors to fully immerse in the moment [19].

This paper focuses on transitioning from technological to philosophical perspectives within AI interactive systems, enhancing user experience by designing immersive output modes.

### 2.2. Multi-Person Physiological Data Interaction

Biological data, collected via wearable biosensors, records human physiological signals detected by biological materials. This data is then processed into a format suitable for experimental research [6]. Marshall McLuhan distinguished “hot” from “cool” media: hot media are rich in information and require minimal audience engagement, whereas cool media offer less detail and demand greater participation and interpretation from the audience [20]. Advancements in biosensor technology have transformed physiological data into a communicative bridge between humans and computers, introducing a novel medium that facilitates co-creation among designers and users.

EEG offers a non-invasive approach to monitoring brain electrical activity by placing electrodes on the scalp to record the brain’s spontaneous electrical signals over time [21]. EEG data serves as a physiological medium, with popular commercial devices such as Muse, Emotive, and NeuroSky facilitating its collection.

Multi-user EEG data refers to the real-time collection of brainwave data from multiple participants. As shown in Table 1, this study summarizes multi-user EEG-based works and analyzes them according to interaction methods, types, and performance. The use of multi-user EEG data in interactive works enables more complex feedback mechanisms, enhancing playability. The interaction systems typically involve either cooperative or competitive modes, but regardless of the control method, BCI (brain-computer interface) interactions tend to generate positive user experiences. Social and emotional interaction levels are notably higher during BCI control [22].

Social interaction environments, essential for multi-user collaboration systems, significantly influence individual experiences, including presence and immersion [23]. Thus, combining individual user experiences with social interaction creates a communal experience. For example, David Rosenboom’s “Alpha Checkers” allowed two players to interact in a visual environment by controlling the game board through alpha waves [24]. Other works such as “New York Biofeedback Quartet” [24], “Ecology of the Skin” [24], MoodMixer [25], Ringing Minds [26], and “Portable Gold and Philosophers’ Stones” explored collective brainwave influence on music generation, highlighting cooperation between multiple individuals [24]. Additionally, “Vancouver Piece” examined shared identity through enhanced lighting effects in front of a mirror, emphasizing the collaborative experience of multiple users [24]. Jacqueline Humbert’s “Alpha Garden” demonstrated how synchronized alpha waves from participants could enhance control over a watering system, showing how cooperation influences the experience [27]. Similarly, “Brainwave Etch-A-Sketch” allowed participants to collaboratively draw by tracking their brainwaves [27]. “Hopscotch—a mobile opera for 24 cars” [24] was a performance where brainwave signals from audience members were collected to generate a responsive operatic scene within a limousine. “Assembly Cognogenesis” [28] created a virtual reality environment where two participants used neural and gestural interfaces to interact with an artificial life world, requiring them to cooperate to evolve entities within the virtual space. Lastly, Suzzane Dikker’s “Mutual Wave Machine” used EEG activity and facial expression visualizations to display thought synchronization, exploring the neural and emotional connections between people and emphasizing the deeper levels of cooperation and communication among individuals [29]. This form of interaction not only enhanced user experience but also fostered social cooperation and engagement among participants, showcasing the potential of BCI technology in promoting multi-person collaboration.

In addition to cooperative approaches, researchers have delved into competitive interaction systems within media. “Bacteria Hunt,” a multi-person EEG-based game, utilizes alpha waves and Steady State Visually Evoked Potentials (SSVEP) for point competition [30]. This game explores interactive player control mechanisms in competitive settings by integrating relaxation and attention-related brainwave frequencies, underscoring the significance of collective participation. “Brainball” enables participants to navigate a steel ball through a race using physiological signals from EEG and Electromyography (EMG) [30]. “NeuroBrush,” a web application, facilitates a competition in creating postmodern art through BCI inputs [31]. The platform fosters competition and social cooperation by enabling real-time sharing of the art-creation process and dynamic background color adjustments, enhancing the creative experience.

Beyond multi-user EEG data, various biological data types also enable individual interactions. For instance, Lozano-Hemmer’s “Zoom Pavilion” [32] dynamically projects participants’ facial and movement data in real time, captured within the installation space [33]. George Zisiadis’s “Pulse of the City” [34] captures real-time heart rate data when participants grip a heart-shaped object’s handles, transforming it into musical beats via integrated speakers. Misha Sra and colleagues [35] created interactive pieces using breath as a direct control mechanism, employing the Zephyr BioHarness sensor for four distinct control actions.

A growing body of research utilizes physiological data devices and methodologies to enhance participant interactions. Evidence indicates that such approaches heighten audience engagement and attention while broadening the system’s sociability via intricate and structured feedback mechanisms. This project seeks to extend these foundational studies by investigating the integration of multi-user EEG data into artificial intelligence systems.

### 2.3. Integration of EEG Data in AI Systems

Digital media are integral to systems, and the ideology along with the way we interact with digital media works are often found in the way their structure operates [36]. Integrating EEG data into AI systems requires deconstructing its output, input, and data-processing phases. Understanding these computational processes is essential to discerning how the system creates representations [36]. However, as technology evolves, the distinctions between various media are becoming increasingly indistinct, leading to a convergence. Recently, researchers have focused more on technical aspects rather than critically examining how media integration into new systems could improve their experiential quality.

Physiological data enhances AI interaction systems in three key dimensions.

Initially, physiological data interfaces stimulate unique sensory and cognitive modes in AI system users, including vision, hearing, spatial awareness, and EEG inputs. As shown in Table 2, this study lists various AI systems and the types of input data used.

Visual input is a key process in AI systems, where cameras capture environmental data, providing rich information about users’ appearance. In the “AI Nüshu” project [37], two AI agents observed the environment and recorded audience behavior, integrating it with traditional Nüshu poetry to showcase AI’s potential in language generation. Similarly, “Cangjie’s Poetry” [38] relied on visual input, while “Transferscope” [39] allowed users to capture and transform images, seamlessly integrating them into new scenes. Auditory input, using text and speech conversion, enables AI to perceive user voices. In the Mayfly installation, voice-generated content explored linguistic taboos and censorship. A museum recommendation system [40], powered by a fine-tuned GPT-4 model, provided personalized recommendations based on user input and contextual factors like location and visit time. The “Storyteller” prototype [41], developed by The Met, Microsoft, and MIT, highlighted AI’s role in cultural heritage preservation. Recent studies are expanding AI perception. In Attracting Museum Visitors Through AI-Generated Narration and Gameplay, Kinect sensors and spatial data enhanced user interaction for a more immersive experience. “New Nature” [42], a digital bio-park by Marpi, used sensors to capture audience physiological data, which influenced immersive projections, creating surreal visual effects. In “Wu Xiang Zhi Xiang”, participants wore EEG devices that influenced the visuals on screen, reflecting brainwave activity [43].

Secondly, physiological data as a medium introduces abstract things (such as emotions) into the system, providing communication opportunities for multiple participants. The audience’s inner experience (emotions, thoughts) becomes externalized through the medium, thereby affecting social interaction. For instance, Mariko Mori employs EEG devices to record audience brainwaves across Delta, Theta, Alpha, Beta, and Gamma bands. These are then algorithmically translated into emotional values, enabling viewers to recognize their and others’ emotional visual patterns. In “Cacophonic Choir”, clear story comprehension requires visitors to lean in closely to an AI agent. Such physical proximity is designed to enhance empathy among participants [18].

Ultimately, physiological data enhance conceptual articulation within AI systems, fostering ideological reflections and dialogues among users. Artistic concept expression enriches the work, accentuating its theme via metaphors and multifaceted narratives. In Meshi A’s installation, participants engage with a machine learning classifier that analyzes their physical traits and movements in real time, juxtaposing these with prerecorded animated characters [44]. Beyond technical exhibition, this interaction prompts audience reflection on collective memory and identity formation. Rafael Lozano-Hemmer’s “Nivel de confidence” addresses Mexico’s issues of violence and missing persons via digital technology and facial recognition mechanisms [32]. The installation bolsters collective awareness of national memory lapses utilizing biometric monitoring algorithms.

Present studies on incorporating physiological data into AI multi-person systems reveal a deficiency in systematic approaches and methodologies, hindering the formation of profound connections from a media standpoint. This challenge primarily arises from technological instability, the analytical complexity of data, and the intricacies of merging diverse media forms. In this context, our study aims to investigate the integration of EEG data into AI interaction systems—covering data input, processing, and output—to improve user experience.

## 3. Prototype

This research employs an interactive installation to explore the enhancement of AI system experiences through multi-person EEG data across various interaction modalities. This methodology of this project is practice-oriented, focusing on design of the system prototype and engaging with contemporary practical applications and scholarly discussions within the field. This study adopts an interdisciplinary approach, integrating theories from computer science, biology, communications, and psychology.

**Figure 4:**
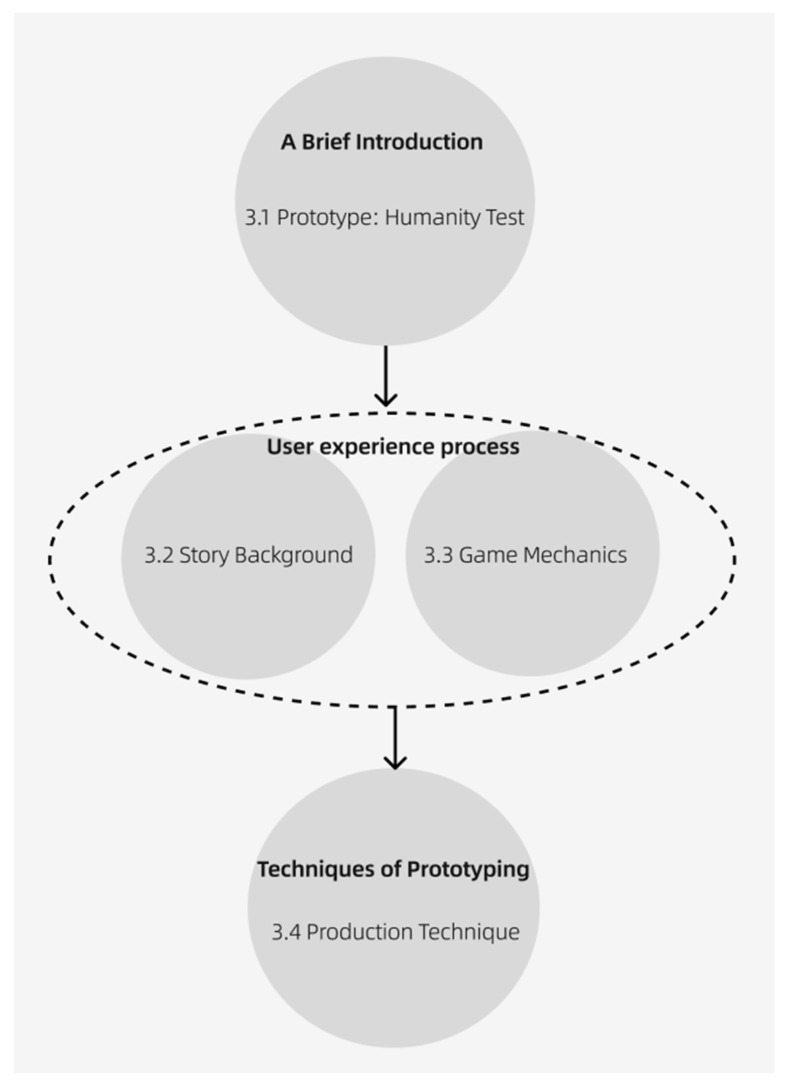
The general vision of Section 3.

As shown in the vision map below, the prototype consists of four parts: 3.1 A brief introduction; 3.2 Story Background; 3.3 Game Mechanism; 3.4 Production Technology. See Figure 4.

### 3.1. Prototype: Humanity Test (Voight-Kampff 2.0)

The “Humanity Test” embodies a sci-fi-themed interactive installation. This project integrates multiple media forms to deliver gamified narrative experiences, which take approximately 20 min, necessitating cooperation between two participants, where engaging enhances experiences from each other.

**Figure 5:**
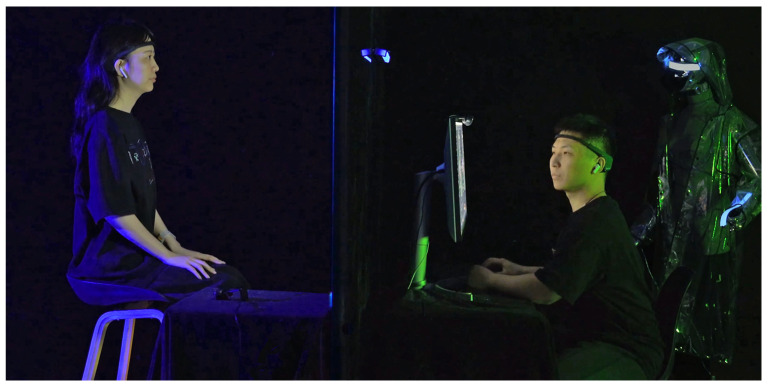
Voight-Kampff 2.0 on the exhibition.

Participants engage according to predefined rules, with their real-time EEG data influencing the system’s overall ambiance. Initially displayed at the Beihang University Art Exhibition Hall, the installation was later featured at the SIGGRAPH Asia Art Gallery [45] and an art museum in Chengdu, Sichuan, attracting a diverse audience (see Figure 5).

The installation’s thematic inspiration originates from the science fiction novel “Avalanche”, which envisions a scenario where computer viruses can affect the human cerebral cortex, turning individuals into mechanized entities capable only of executing programs input by others. As described by the author Wiener, it’s a human nightmare where individuals are confined within machinery bounds, without autonomous consciousness, being used to serve machines. Consequently, this project provokes ongoing reflection on questions: Can humans become machines? Where is the boundary between humans and machines?

### 3.2. Story Background

The installation is inspired by the Voight-Kampff machine from the *Blade Runner* novel. This work tells a science fiction story set in a futuristic central city. Amy, a psychologist working for an AI company, specializes in distinguishing between humans and AI. In this future, AI is designed to closely resemble humans but remains flawed in emotional processing. Amy’s latest task is to use the Voight-Kampff 2.0 machine’s emotion-recognition technology to determine whether her counterpart is human or AI. Although AI has made significant progress in simulating emotions, does it truly possess self-awareness? In this piece, two participants take on the roles of Amy and the test subject, as shown in Figure 5. Using the installation, they perform their respective tasks, engaging in a gamified narrative experience. In the following text, we will refer to Amy as the questioner and the test subject as the respondent.

### 3.3. Game Mechanics

**Figure 6:**
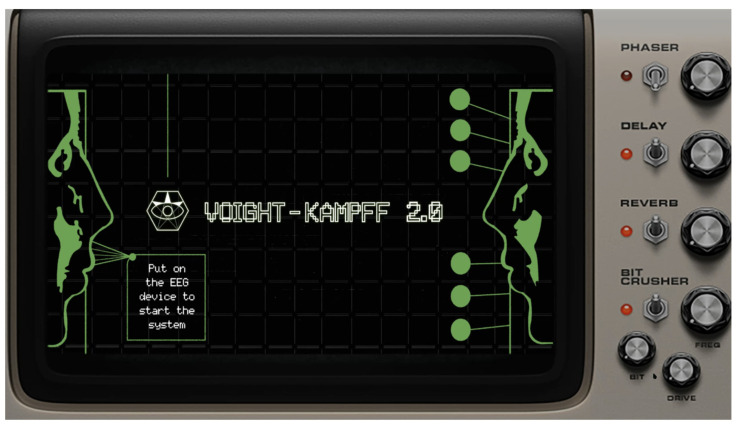
The startup interface of Voight-Kampff 2.0.

The experimental setup entails a collaborative interaction between two participants who wear EEG devices, headphones, and cameras. They cannot communicate nor see each other directly. Figure 6 shows the startup interface, which includes the title of the work and operational instructions. When the questioner follows the instructions, puts on the device, and their focus level reaches the threshold, the system will automatically start.

**Figure 7:**
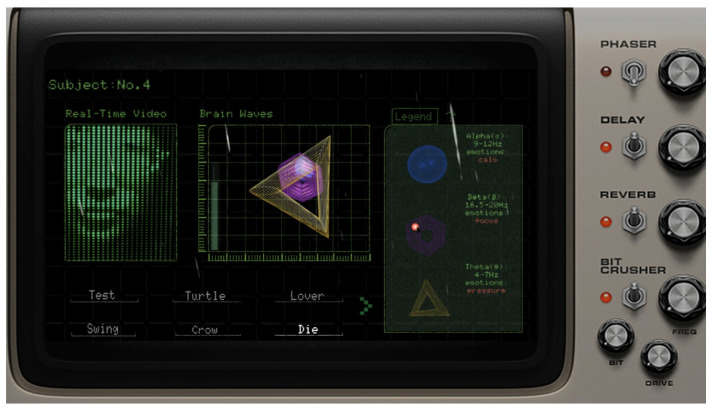
The interface viewed by the questioner.

Figure 7 shows the interface viewed by the questioner, which is composed of four sections: the respondent’s real-time facial expressions, the visualization of EEG data, the “Human Nature Test” questions, and an EEG legend with operational guidance. The EEG visualization includes the user’s alpha, beta, and theta waves, represented by a blue sphere, a purple hexagon, and a yellow triangle, respectively. As these wave frequencies increase, the corresponding shapes enlarge.

**Figure 8:**
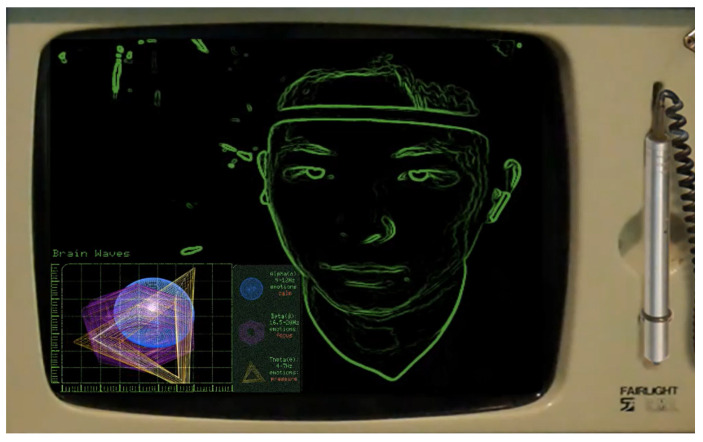
The respondent’s interface.

As shown in Figure 8, the respondent’s interface consists of two parts: the “Human Nature Test” questions and the visualization of the respondent’s EEG data.

**Figure 9:**
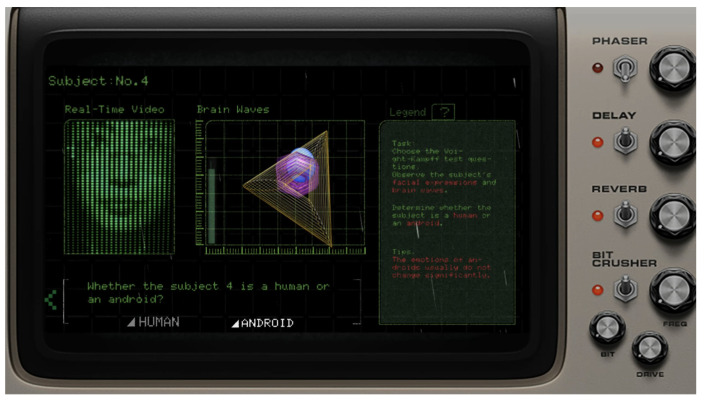
The questioner makes their selection through the interface.

When the questioner clicks on a “Human Nature Test” question, the respondent hears and sees sound and visuals designed to provoke emotional reactions (as shown in Figure 9). The designed trigger questions include three positive and three negative prompts. The questioner then observes the EEG viewer on their interface to determine the respondent’s identity: Is it a human or a virtual being? The basis for judgment includes noticeable feedback in the EEG signals for real humans, whereas virtual beings, despite mimicking human facial expressions and voice responses (detailed in the fourth module of the system in Section 3.4), show no EEG fluctuations. Finally, the questioner makes their selection through the interface shown in Figure 9.

### 3.4. Production Technique

**Figure 10:**
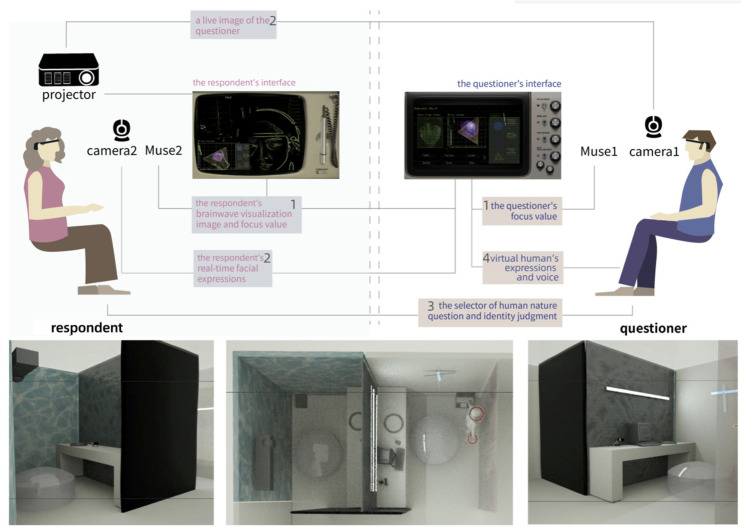
System map.

As shown in Figure 10, our project integrates software and hardware design through an interactive system to provide a comprehensive user experience environment. The hardware setup comprises a display screen, headphones, EEG devices (Muse), cameras, a mouse, a computer, and a projector.

The rendered image in the lower half of Figure 10 shows how acrylic panels are used to divide the 3 m × 5 m space into two smaller areas, allowing the questioner and respondent to experience the installation separately. A 3 m × 3 m transparent acrylic board at the rear, blue and green fluorescent strips decorating the space, a 3 m × 5 m mirror floor covering, two black podiums, and two chairs each positioned on one side of the space, with a speaker underneath the podium.

The upper half of Figure 10 illustrates that the system is composed of four modules:

1. EEG data transmission and processing;
2. Facial data transmission and processing;
3. Mouse-based interactive question selector;
4. Virtual character’s facial and voice feedback.

**Figure 11:**
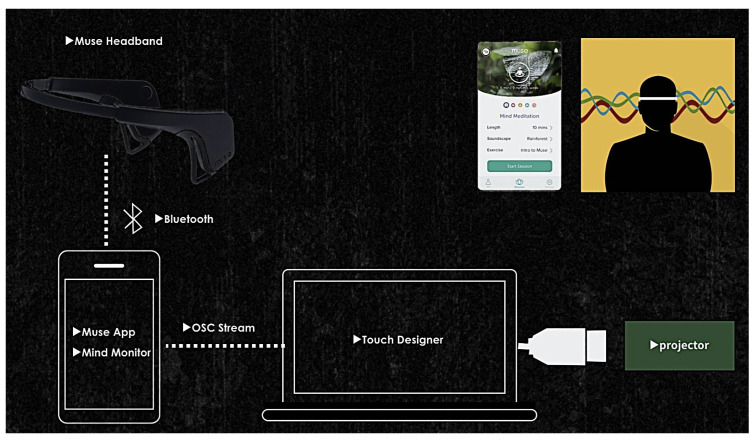
How EEG data is processed.

Firstly, Figure 11 illustrates how EEG data is processed using the Muse Headband, which captures brain activity and communicates it wirelessly via Bluetooth to a smartphone app like the Muse App (version 3.8.2) and Mind Monitor (version 2.1.1). These apps process the EEG signals, providing real-time feedback on brain activity. The data is then streamed using OSC to Touch Designer (version 2022.31000), a desktop application that further processes the signals into dynamic visualizations with the help of custom Python scripts (version 3.9.7). The visualized data is projected through a projector, allowing for real-time display of brainwave activity.

The second module captures facial data via a camera. The third module enables the playback of emotion-triggering environmental sounds and visuals through mouse clicks, also implemented using Python (version 3.9.7). In the fourth module, when the questioner selects a question, it is sent via an API to a large language model (ChatGPT). The ChatGPT API, provided by OpenAI (version 4.0), allows developers to integrate large language models into local applications. The large language model returns relevant text, which is processed by the local program and converted into speech using text-to-speech technology to generate the virtual character’s response.

## 4. Experiment

This study identifies the device version (specifically, game mechanics) as the independent variable, with user experience constituting the dependent variable. Regarding questionnaire responses, individual participants represent the unit of analysis; in contrast, pairs of participants are analyzed as a single unit in interviews.

The aim of this experiment is to investigate whether EEG signals can influence interaction with an AI system and enhance the user experience. In the experimental group (C1), participants interacted with the AI system through EEG devices, allowing the AI to receive and utilize EEG signals to adjust the interaction or experience. In the control group (C2), participants interacted with the AI system without EEG input, with the system responding solely through traditional inputs, such as a mouse.

To minimize the influence of external factors (such as visual, auditory, environmental settings, and individual differences) on participants’ immersion and emotional experience, we applied the control variable method. This involved standardizing experimental conditions, such as controlling lighting, sound levels, and temperature. For example, we used the same screen and sound intensity and conducted the experiment at the same time each day to reduce environmental variability. Additionally, we implemented random assignment to distribute participants across different experimental conditions, ensuring that variables like gender, age, and experience were evenly balanced, thereby minimizing their potential impact on the study’s outcomes.

### 4.1. Experimental Procedure

Before the experiment, participants received training using the Muse App (version 3.8.2) included with the EEG device to familiarize themselves with its controls. EEG signals were recalibrated before each session to ensure accuracy. Subsequently, participants were equipped with physiological sensors and given brief instructions on how they work. They were suggested to engage in meditation or focus on a specific thought to enhance concentration. We also provide a concise introduction to the device’s background story. Throughout the experiment, video recordings and game-completion times were documented as supplementary metrics to evaluate their experiences.

**Figure 12:**
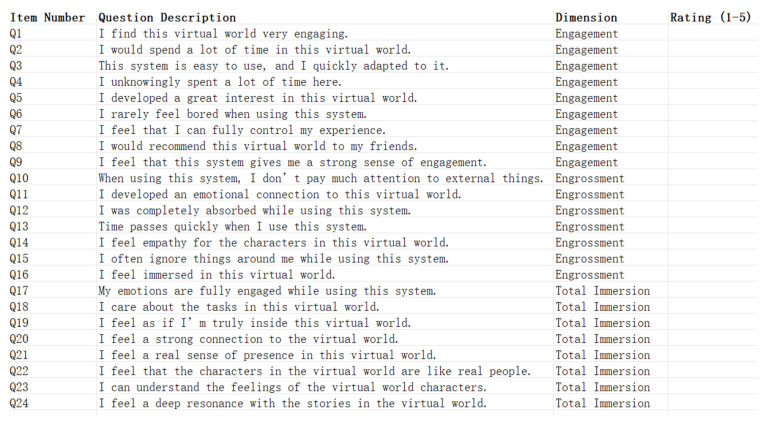
Game Immersion Questionnaire (GIQ) was designed with 24 questions to assess various aspects of game immersion. Each dimension is measured using a 5-point Likert scale to gauge users’ experiences.

Following the experiment, participants were asked to complete the Game Immersion Questionnaire (GIQ) and the Self-Assessment Manikin (SAM) to gauge subjective immersive experiences. The Game Immersion Questionnaire (GIQ) is designed to measure immersion in game-based virtual worlds [46]. Its primary aim is to assess the level of immersion experienced by users in games or similar interactive systems across three dimensions: engagement, engrossment, and total immersion. Each dimension is measured using a 5-point Likert scale to gauge users’ experiences, as shown in Figure 12.

**Figure 13:**
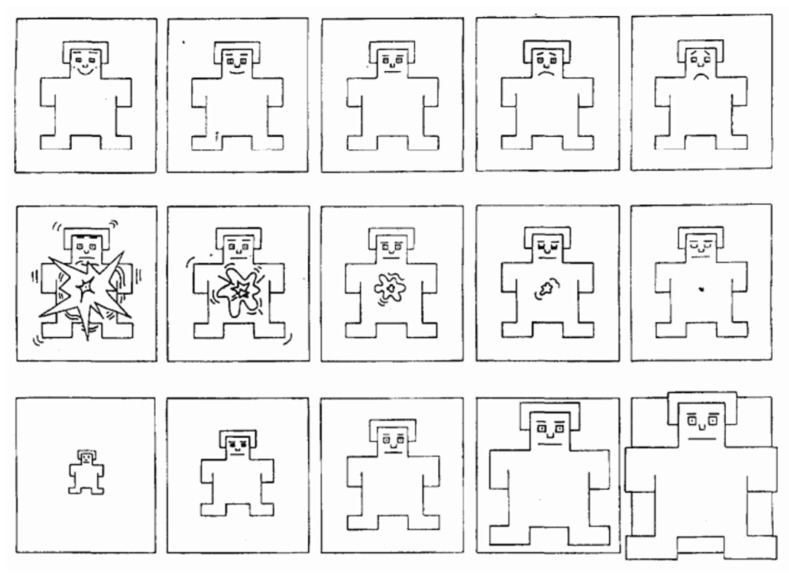
SAM’s use of pictures to communicate the scale.

The Self-Assessment Manikin (SAM) is a questionnaire used to assess emotional responses, measuring individuals’ emotional states during specific interactions or experiences. It evaluates emotional reactions based on three core dimensions: valence/pleasure (ranging from positive to negative), perceived arousal (ranging from high to low), and dominance/control (ranging from low to high) [47]. SAM uses images to convey the scale, and due to its non-verbal design (see Figure 13), the questionnaire is accessible to individuals regardless of age, language proficiency, or educational background.

Subsequent to administering the questionnaire, we conduct semi-structured interviews with participants to delve deeper into their experiences with the various mechanisms while upholding their privacy.

### 4.2. Participants

To ensure diversity and a broad participant base, we employed multiple recruitment methods to find suitable participants. Recruitment channels included posting information on social media and referrals from previous participants. The participants were aged between 20 and 40, with 10 females and 10 males.

To maintain experimental control and validity, participants were divided into two groups: five pairs tested the first version (C1), and five pairs tested the second version (C2) as a controlled experiment. All participants had normal or corrected vision and used computers daily, possessing a certain level of computer proficiency. While most participants had no prior experience using EEG devices, three participants indicated they had previously worn EEG data measurement devices.

To encourage participation, all participants voluntarily took part in the study and signed informed consent forms. As compensation for their involvement, we also promised a monetary reward to express our gratitude.

### 4.3. Ethics and Procedure Documentation

In this study, we strictly adhered to ethical standards to ensure that participants’ rights and privacy were fully protected. All participants voluntarily signed informed consent forms prior to the experiment, fully understanding the study’s purpose, procedures, potential risks, and data usage. To protect privacy, all collected EEG data and feedback questionnaires were anonymized, with personal identity information stored separately from the data. The data was used solely for research purposes and stored on encrypted servers, accessible only to authorized researchers. Due to the limited sample size in our experiment, the EEG data collected was insufficient to support comprehensive analysis. Therefore, we opted to prioritize questionnaires and interviews as our primary research methods. Upon the completion of the study, the EEG data was securely destroyed in accordance with data protection protocols, ensuring that it will no longer be used or disclosed. Throughout the study, we ensured participants’ comfort and safety, and the experiment could be paused at any time if discomfort arose. While participants had the option to withdraw at any point, no one chose to do so during the process.

## 5. Results

We employed a specialized statistical method (non-parametric techniques) to analyze the participants’ rating data, while also organizing the interview responses into three main themes. First, we presented the results related to immersion and emotional experience scores, followed by a discussion of participants’ understanding and feedback on the work.

### 5.1. Spatial Immersion

**Figure 14:**
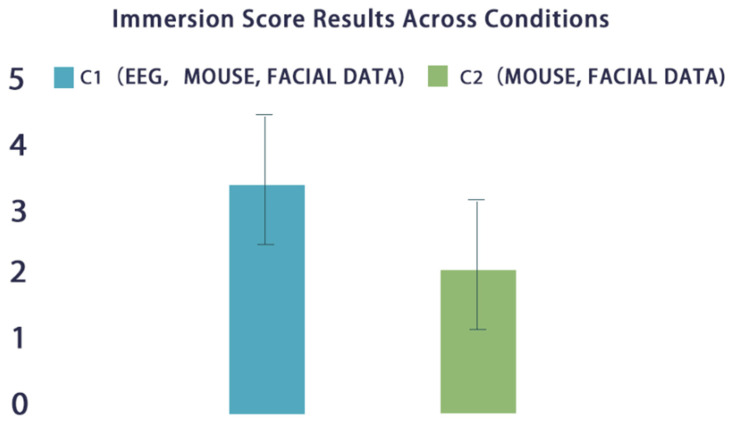
GIQ scores for different mechanisms. The choice of the 0–5 scale was due to the design of the GIQ, where each question was scored on a Likert scale (0–5).

Figure 14 displays the average scores of the Game Immersion Questionnaire (GIQ) under two different conditions (C1 and C2). These scores were obtained from participants’ responses to the questionnaire. By comparing the average scores under the two conditions, it is clear that their immersion scores were higher when using EEG data.

The Wilcoxon signed-rank test result shows Z = 2.1, indicating a significant difference between the conditions with and without EEG data. A *p*-value of less than 0.05 (*p* < 0.05) demonstrates statistical significance, meaning that this difference in immersion scores between using and not using EEG data is very likely not due to chance, but rather caused by the condition itself (whether EEG data was used). See Figure 14.

Post-experiment, 15 participants highlighted EEG data visualization and sonification as pivotal in amplifying immersion levels. “The sensor gave me a greater sense of participation in the game” (P2, Female). Eight participants who enjoyed the EEG control aspect commented that using their mind to trigger actions made the game more novel and engaging, enhancing its playability. This sense of participation and enjoyment seemed particularly strong when the screen effects were “controlled by my thoughts, not by a mouse” (P19, Male).

Additionally, seven participants noted that the integration of spatial design with EEG data, such as weak control of ambient lighting through brainwaves, effectively enhanced their immersive experience to some extent. “The lighting arrangement reminded me of *Blade Runner*; I felt like I had entered a scene in the game, becoming a game character”, (P4, Female). However, four participants mentioned that their sense of immersion was slightly diminished at times due to connectivity issues with the equipment.

### 5.2. Emotional Experience

Table 3 below presents the mean Self-Assessment Manikin (SAM) scale scores for both mechanisms.

This table shows the participants’ scores on the three emotional dimensions—valence, arousal, and dominance—under the C1 and C2 conditions. The valence (3.8) and arousal (4) scores in the C1 condition were significantly higher than those in the C2 condition (valence 1.9, arousal 1.9), indicating that participants’ emotional experiences were more positive and intense in the C1 condition, while the C2 condition elicited more negative and calmer emotional responses. However, the dominance score was higher in the C2 condition (3.8) compared to the C1 condition (2.4), suggesting that participants felt a greater sense of control in the C2 condition, despite the less intense emotional experience. Overall, the C1 condition provided a richer emotional experience, but with less perceived control, whereas in the C2 condition, participants had a stronger sense of control, though their emotional experience was less engaging.

To gain deeper insight into participants’ emotional perceptions under EEG control, we inquired whether utilizing their consciousness to manipulate the game enhanced their awareness of their own and their collaborator’s emotional states.

“Beyond creativity, what truly moved me was how the interaction takes you deeper into yourself. Seeing your EEG waves creates strong emotions and enhanced teamwork with your partner.”

(P6, Female)

“This game made me reflect on my collaborative relationship with others.”

(P17, Male)

Some participants experienced “strong emotional resonance” (P10, Female), especially in the segment where they had to interact with their partner.

Twelve participants acknowledged the efficacy of EEG data in reflecting emotional states, especially through the mapping of colors and shape transformations. Concurrently, two respondents expressed a desire to “see a wider variety of emotional states captured and displayed by EEG data” (P10, Female). Another participant added: “Emotional visual feedback is sometimes not clear and has a delay.“ (P9, Male).

### 5.3. Conceptual Communication

**Figure 15:**
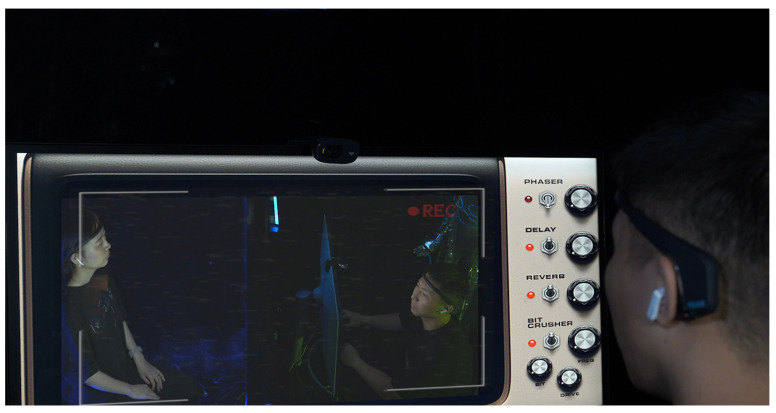
Experimental process.

In the interview phase, inquiries regarding the efficacy of thematic communication were posed (see Figure 15). A considerable number of participants affirmed that the project adeptly communicated its themes concerning humanity and technology. Many respondents were particularly captivated by the exploration of “self-awareness” and “emotion” themes within the experience.

“The interaction between Amy and Lia showcased the limitations of artificial intelligence in understanding human emotions.”

(P11, Male)

“I feel that the UI design complements the project’s theme, enhancing the overall sense of the experience.”

(P5, Female)

“It made me rethink the role of artificial intelligence in our lives.”

(P14, Male)

“This project is an enlightening work, especially in an era where everyone extols technology.”

(P20, Female)

“This work is a reflection on the sustainable development of future AI technology.”

(P19, Male)

In conclusion, informal interviews predominantly affirmed the project’s efficacy in articulating complex concepts via immersive experiences, resonating with users both emotionally and cognitively.

## 6. Discussion

Building on the results from the previous chapter, we further explored these key findings and discussed the optimal integration of physiological sensors into traditional control systems. As summarized in Table 4, we identified three design directions for AI-driven multi-person interaction systems using EEG data: enhancing immersion, creating emotional experiences, and facilitating conceptual expression. Additionally, we discussed the limitations of our work and proposed opportunities for future research.

### 6.1. Creating Spatial Immersion

Many participants mentioned that they felt a higher degree of involvement when using EEG interaction, attributing it to the perceptual origins of spatial immersion. This phenomenon is because spatial immersion primarily originates from the perceptual level, and visualizing and sonifying biometric data are common methods for perceptual-level design based on experience.

**Figure 16:**
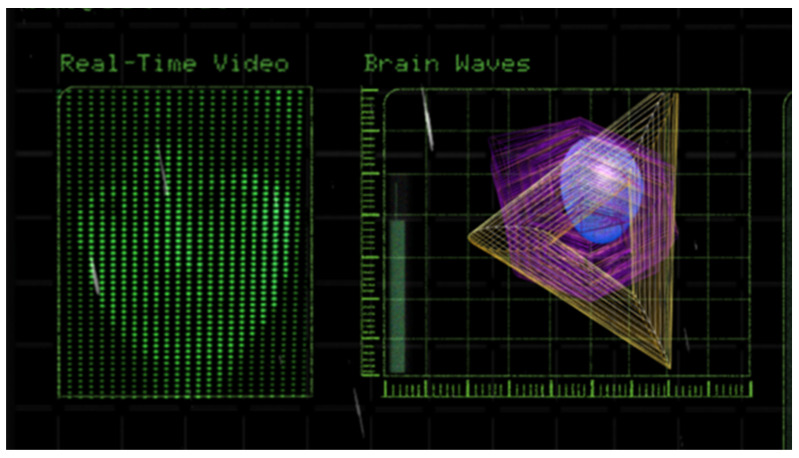
Audio-visual effects design.

Design considerations encompass three main aspects: input data, data processing, and output content, focusing on the mapping between EEG data and immersive audio-visual effects. Mapping EEG data for immersive audio-visual effects involves selecting frequencies and bands aligned with artistic objectives. For example, in this study, the EEG bands selected for analysis were primarily alpha, beta, and theta. Alpha waves (8–12 Hz) correspond to relaxation and calmness; beta waves (13–30 Hz) to alertness and stress; and theta waves (4–7 Hz) to deep relaxation and concentration [48] (see Figure 16).

Following data collection, biometric information from biological sensors undergoes processing to align with creative specifications, utilizing ChatGPT (version 4.0). For example, in this research, pre-labeled EEG data served as the training data set, facilitating the generation of specific sequences. A machine learning-based emotional model was developed through the iterative accumulation and categorization of data. Subsequently, the model’s accuracy underwent validation through cross-validation techniques.

The design of audio-visual effects relies on thematic alignment and an understanding of the data’s inherent meanings and characteristics. For instance, alpha, beta, and theta brainwave frequencies within specific emotional model ranges trigger corresponding changes in emotional graphics. The three fundamental symbols act as linguistic signs for abstract concepts, reflecting Saussurean semiotics principles. For instance, the triangle inherently conveys an image of stability, and the circle conveys an image of calm, soothingness, etc. Post-mapping and dynamic input data lead to continual modifications in the work’s presentation, including screen transitions and variations in graphics and sound, such as screen transitions, changes in graphic size, color changes, changes in quantity, changes in sound pitch, etc. Therefore, the transition between different states needs to be smooth and sensitive to ensure well-timed feedback and a good experience for the audience. Our research indicates that introducing a deliberate delay in data processing can result in a more gradual input data transition, which is particularly beneficial for abstract visual effects like singular shapes. In this project, we used Python’s “time.sleep()” (version 3.9.7) function to impose a predetermined delay, effectively moderating the pace of the final three graphical transitions.

Furthermore, there are intricate connections between auditory feedback design and narrative structure. For instance, audience-selected questions trigger specific questions and ambient sound responses. Environmental sounds from the International Affective Digitized Sounds (IADS) library are employed for humanity-related inquiries, activating upon question selection to evoke experiencers’ emotional responses.

In the integration of EEG data with spatial immersion experiences, not only visual and auditory changes are involved, but there is also potential to introduce haptic feedback. For example, as brainwave patterns change, haptic feedback devices (such as vibration or airflow) can synchronize with these changes, enhancing participants’ perception of the virtual world. In future designs, multi-sensory integration will be crucial for enhancing spatial immersion experiences. By combining tactile sensations, temperature variations, or other physical feedback, virtual experiences can be elevated to a more immersive and realistic level.

Additionally, in the realm of biological data processing, current EEG analysis primarily focuses on specific frequency ranges (e.g., alpha, beta, theta waves). Future research could explore more brainwave features, especially when combined with other physiological data such as heart rate or skin conductance, to enhance the system’s understanding of users’ emotions and states. Through multi-modal biological feedback, designers can create more refined and dynamic immersive experiences based on participants’ psychological and physiological conditions.

### 6.2. Creating Emotional Experiences

Incorporating participant feedback proved that artworks fostering immersive emotional experiences are more memorable. Our findings suggest that AI systems enhanced with EEG data not only facilitate interaction but also promote empathy, capturing significant attention and leaving a lasting impact on individuals.

**Figure 17:**
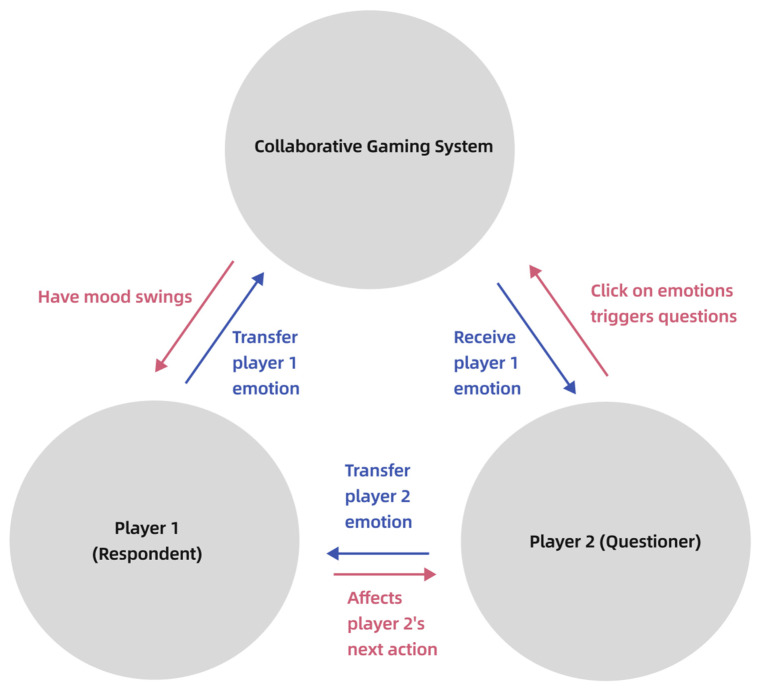
Emotional loop.

This project specifically explores the synergy of shared emotional experiences and social enjoyment among participants. By melding individual immersive experiences with social interactions, a collective experience emerges, enriching each participant’s journey (see Figure 17). The design fosters a collaborative experiential mode, where participants’ concurrent EEG data use propels a common objective. The system processes one participant’s data to influence the experiential state and content of another, who then adjusts their responses based on their partner’s feedback. Such collaboration underscores the system’s holistic functionality, as depicted below.

Analysis of participant performance and experimental data indicates that collaboration tends to elicit stronger emotions, impacting EEG data readings. These readings subsequently influence the counterpart’s emotions and the interaction’s collective outcome. Furthermore, expressing emotions fosters social interactivity, creating a more seamless shared experience. Consequently, emotions effectively circulate between the two participants, creating a dynamic interplay.

In the creation of emotional experiences, in addition to collaboration and emotional resonance, the real-time nature and accuracy of emotional feedback are crucial factors affecting the quality of the experience. Future research could explore faster emotional feedback mechanisms to ensure that systems can promptly capture and respond to participants’ emotional changes. Moreover, emotional resonance is not limited to two-person interactions; it can also be expanded to multi-person environments. By synchronizing multiple participants’ biological data (such as brainwaves, heart rate, etc.), a shared emotional experience system can be established to enhance team collaboration and emotional connectivity. For instance, in multiplayer games or virtual social platforms, participants could use biofeedback to sense others’ emotional states and adjust their interaction accordingly.

Furthermore, the visualization of emotional experiences is not confined to images and sounds but can also be expressed through the behavior and expressions of virtual characters. For example, when participants’ brainwaves reflect emotional changes, virtual characters can respond with corresponding behaviors, such as changes in facial expressions, body movements, or vocal tones. This more anthropomorphic form of feedback can strengthen emotional transmission, making the empathetic experience in virtual worlds more authentic and profound.

### 6.3. Ideology

In this section, we delve into the expression of concepts and the employment of specific strategies, aiming to transcend the technicism discussed previously. Our design approach integrates spatial effects and interactions with a deep exploration of “meaningful creation” concerning AI technology themes. The objective is to intertwine human and technological discussions with the brain interactions of participants, thereby enriching the narrative.

**Figure 18:**
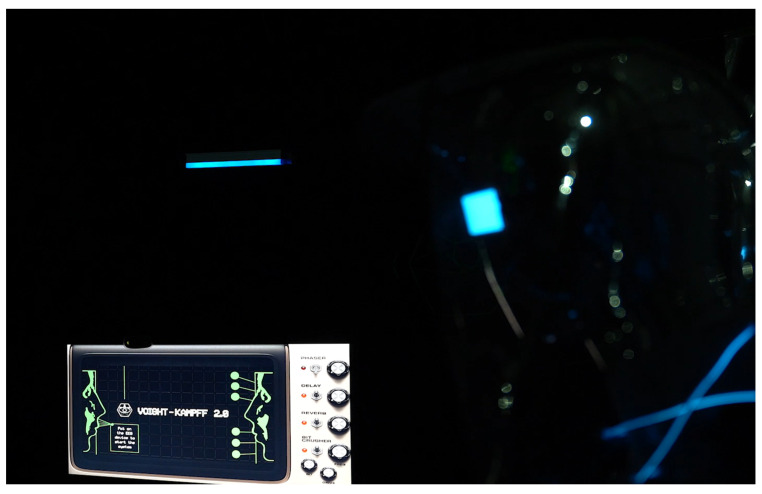
Ideological design.

Leveraging speculative design, we envision future lifestyles via prop design and scene construction, crafting immersive storytelling that uncovers human dilemmas and opportunities, thus vividly conveying thematic ideas. By conceptualizing a scenario and narrative around a humanity test, we intend to provoke thought on the distinct nature of humans versus machines—centering on emotions and consciousness. Conserving autonomous consciousness emerges as a pivotal concern in future human-machine dynamics. Therefore, “self-awareness” and “emotions” stand at the forefront of our project, shaping the primary medium of interaction that mirrors individual emotional fluctuations through EEG data. The AI system’s virtual persona acts as a third entity within the space, embodying a data-driven intangible personality. This setup encourages reflection on human subjectivity and the evaluation of objectivity, as illustrated in Figure 18.

This project explores diverse narrative possibilities. Initially, the introduction of AI technology catalyses the shift in interactive artworks from relying on static, pre-written programs to incorporating dynamic, self-learning algorithms. This transition allows the AI to analyze audience interactions, subsequently adapting and formulating new response patterns in real time. Conversely, in experiences involving two participants, the differing identities provide varied experiential perspectives, imbuing the artwork with a range of narrative potentials.

Regarding the issue of “human subjectivity”, future research can further explore the externalization of self-awareness and its integration with technology. As artificial intelligence and biofeedback systems advance, human emotions, behaviors, and even thoughts may increasingly be externalized as data, posing new challenges to human autonomy. How to maintain human subjectivity in this technological environment, avoiding manipulation or over-reliance on technology, is a social issue that warrants further study. Designers can introduce more reflective interactions, allowing users to become aware of how their emotions and behaviours can be stored as data and even be manipulated, thereby encouraging deeper reflection on human-machine relationships.

Additionally, speculative design will continue to hold significant potential for application in the future. By constructing virtual and futuristic scenarios, people can better understand the role of AI and biofeedback technologies in future societies. For instance, by designing interactive works with multiple outcomes, users can see different directions in the development of technology and human nature based on their choices. This diversity not only enhances the interactivity and engagement of the experience but also provides users with more space for reflection on the ethical and moral issues brought about by technological advancements.

### 6.4. Limitations

Although this study represents an initial exploration, its generalizability is constrained. Firstly, our focus was on particular AI systems and experiential mechanisms, implying that alternate choices could yield different user experiences. Secondly, exhibition time and spatial constraints restricted participant numbers and the breadth of experiences. A greater familiarity with the equipment might unveil other outcomes. Thirdly, the current EEG data visualization is confined to simplistic abstract graphics. Future endeavors will aim to develop more scientifically precise and aesthetically appealing data mappings and interfaces.

Additionally, the study did not explore the long-term effects of using EEG data to enhance AI systems in improving user immersion and emotional experience. The participants, aged between 20 and 40, had varying levels of prior experience with EEG devices, with most having no previous exposure, though three participants had used similar equipment before. This lack of diversity in both age and experience could limit the representativeness of the findings, and thus future research should aim to include a broader and more diverse participant base to improve the generalizability of the results.

In the future, our research will conduct a longitudinal study over a period of 3 to 6 months, allowing for multiple interactions to observe long-term changes in user behavior and emotional responses. The sample size will be expanded to 50 to 60 participants, covering a diverse age range of 20 to 60 years and varying levels of experience with EEG devices, ensuring the diversity and representativeness of the results. Lastly, the study will further optimize EEG data visualization techniques to improve scientific accuracy and user engagement, ensuring effective application of the data and enhanced user experience throughout the research process.

## 7. Conclusions

This study explored the integration of EEG data into AI systems in a multi-person interactive environment. By designing a controlled experiment with two interaction mechanisms, the results showed that real-time capture and analysis of brainwave activity significantly enhanced users’ immersion and emotional interaction experiences. Experimental data indicated a 40% increase in immersion and a 50% increase in emotional interaction for participants using the EEG-based interaction mechanism. Emotional reciprocity was particularly stronger during collaborative tasks, leading to smoother social interactions.

This research also demonstrated the potential of integrating EEG data into AI systems. For example, in gaming and virtual reality environments, EEG can dynamically adjust the environment by monitoring brainwave activity in real time, thereby enhancing user immersion. Additionally, EEG data can be integrated into AI-driven therapeutic tools. By analyzing a user’s brain activity, AI systems can identify stress levels or emotional states, aiding healthcare professionals in better understanding patients’ psychological conditions. In group therapy settings, EEG data can enhance emotional interaction and empathy, fostering greater emotional reciprocity and improving therapeutic outcomes.

However, the widespread application of EEG systems faces several challenges. First, collecting and analyzing brainwave data raises privacy concerns, as users may feel uncomfortable with AI systems accessing and interpreting their emotional states. Second, in real-world, multi-user environments, the accuracy and reliability of EEG data may be affected by external noise and individual variability, limiting the system’s precision and scalability.

Overall, integrating EEG data into AI systems presents great potential. However, to ensure the effectiveness and widespread adoption of these systems, it is necessary to address technical, ethical, and societal challenges.

## Figures and Tables

**Table 1:** Multi-agent BCI works summary.

Multi-Agent BCI Works	Participation Mode (Competition/Cooperation)	Type of Work (Interface/Immersive)	Mode of Expression
Alpha Checkers (1969)	Cooperation	Interface	Game
New York Biofeedback Quartet (1969)	Cooperation	Immersive	Bio-music
Ecology of the Skin (1970)	Cooperation	Immersive	Participation experience
Vancouver Piece (1972)	Cooperation	Immersive	Identity exploration
Portable Gold and Philosophers’ Stones (1972)	Cooperation	Immersive	Concert
Alpha Garden (1973)	Cooperation	Interface	Installation
Brainwave Etch-A-Sketch (1974)	Cooperation	Interface	Painting
BrainBall (1999)	Competition	Interface	Game
Bacteria Hunt (2010)	Competition	Interface	Game
MoodMixer (2011)	Cooperation	Interface	Concert
Ringing Minds (2014)	Cooperation	Immersive	Concert
Hopscotch a mobile opera for 24 cars (2015)	Cooperation	Immersive	Interactive drama
Assembly Cognogenesis (2016)	Cooperation	Immersive	Virtual reality
NeuroBrush (2019)	Competition	Interface	Installation
Mutual Wave Machine (2019)	Cooperation	Immersive	Visual display

**Table 2:** Summary of AI systems integrated with physiological data.

AI System	System Input
AI Nüshu (Women’s scripts) (2023)	Visual input
Cangjie’s poetry (2021)	Visual input
Transferscope (2024)	Visual input
Machine Learning in Cyber-Security (2019)	Visual input
Nivel de confidence (2022)	Visual input
New Nature (2018)	Visual input
Ephemera (2024)	Auditory input
Recommendation systems in museums (2023)	Auditory input
Storyteller (2019)	Auditory input
museum AI-generated narration (2022)	Range input
Cacophonic Choir (2014)	Range input
Wu Xiang Zhi Xiang (2021)	EEG input
Wave UFO (2005)	EEG input

**Table 3:** Self-Assessment Manikin (SAM) scale scores of C1 and C2.

	Valence	Arousal	Dominance
C1	3.8	4	2.4
C2	1.9	1.9	3.8

**Table 4:** Summary of the discussion content.

Design Direction	Objective	Strategy	Key Content
6.1 Creating Spatial Immersion	Enhance participants’ sense of immersion.	Data mapping	
	Particularly “full-body” engagement	Data processing	Mapping brainwave bands (e.g., Alpha, Beta, Theta) to audio-visual effects, controlling smooth data changes using programming, symbolic visual design.
		Audio-visual feedback	
6.2 Creating Emotional Experiences	Increase participants’ emotional resonance and social interaction.	Mutual brainwave influence	
	Create memorable and impactful experiences.	Shared goals	Two-person collaboration, where brainwave data mutually influences emotions, generating emotional loops that enhance social interaction and shared experiences.
		Collaborative experience	
6.3 Ideology	Explore the theme of human nature vs. technology.	Speculative design	
	Highlight the distinction between humans and machines through participants’ brainwaves.	Dynamically generated response patterns	Brainwave data showcasing emotional and conscious interaction, symbolizing the reflection on human subjectivity, with AI-driven dynamic interaction responses.
		Multiple narrative possibilities	

## Data Availability

Data are contained within the article.
